# Associations between breastfeeding mode and duration and food neophobia in toddlerhood: A cross-sectional study among Norwegian toddlers

**DOI:** 10.29219/fnr.v64.3615

**Published:** 2020-02-14

**Authors:** Nina Cecilie Øverby, Eli Anne Myrvoll Blomkvist, Elisabet Rudjord Hillesund

**Affiliations:** Faculty of Health and Sport Sciences, Department of Public Health, Sport and Nutrition, University of Agder, Kristiansand, Norway

**Keywords:** breastfeeding, food neophobia, toddlers, introduction of solid food, food fussiness

## Abstract

**Background:**

Research on the association between breastfeeding duration and food neophobia is inconclusive. Breastfeeding and measures to reduce food neophobia are highly recommended to ensure a healthy diet early in life.

**Objective:**

The aim of this study was to evaluate the association between breastfeeding duration and food neophobia in young Norwegian children.

**Design:**

Participants (*n* = 246) were recruited through kindergartens in four Norwegian counties in 2017. The parents of 1-year-olds filled in questionnaires, including standardized questions on breastfeeding and food neophobia. Cross-sectional results are presented. Comparisons of child neophobia score at 16 months of age according to breastfeeding status at various timepoints during infancy were explored in linear regression models adjusted for maternal education and parental food neophobia.

**Results:**

Still being breastfed at 12 months and being exclusively breastfed at 5 months were independently associated with slightly higher food neophobia score at the mean age of 16 months compared to shorter duration of breastfeeding. We found no other associations between breastfeeding duration and child food neophobia.

**Discussion:**

Our study adds to the somewhat scarce literature regarding associations between breastfeeding mode and duration and later food neophobia; some literature shows protective relations between breastfeeding and food fussiness, and others report opposite or null findings.

**Conclusion:**

We found that both being breastfed at 12 months and being exclusively breastfed at 5 months were independently associated with slightly higher food neophobia score at the mean age of 16 months compared to shorter duration of breastfeeding. As the data are derived from a cross-sectional study, these findings should be interpreted with caution.

## Popular scientific summary

- We evaluated the association between breastfeeding duration and food neophobia in young Norwegian children. Food neophobia may be a barrier to healthy eating among toddlers and should be reduced.
- We found that compared to shorter breastfeeding duration, still being breastfed at 12 months and being exclusively breastfed at 5 months were associated with slightly higher scores of food neophobia at 16 months of age.
- Our findings may inform the debate on optimal timing of complementary feeding, but should, given the cross-sectional nature of this study, be interpreted with caution.

Early diet influences a child’s lifelong health and prosperity (1). A diet high in vegetables, fruits, whole grains, and fish yields better health outcomes (2). Food neophobia, meaning reluctance to try new food, is related to a restricted diet with a limited intake of fruits and vegetables at all ages (3, 4). Food neophobia peaks at about 2–6 years of age (5), the age when food preferences develop, and lifelong dietary habits are initiated (6). Food neophobia has a genetic component; however, it can be reduced by parental feeding practices, such as repeated exposure to new and unfamiliar foods and modeling of healthy eating (5). It is important to understand which factors are related to food neophobia early in life in order to improve long-term diet and public health.

In Norway, exclusive breastfeeding is recommended for the first 4–6 months, and continued breastfeeding is recommended for the first 12 months of life (7). Whether breastfeeding per se reduces or increases food neophobia has been discussed (7). The rationale for breastfeeding to reduce food neophobia is that children who are breastfed experience a variety of flavors according to their mother’s diet, giving them a wider exposure to different flavors (8) than those who receive infant formula (9), thereby potentially increasing the child’s willingness to try new foods. The rationale for why breastfeeding should increase food neophobia is the potential delay in exposure to more varied flavors and textures due to breastmilk being a larger component of diet (10). In a review, Cole et al. (10) found no cross-sectional associations between those who are ever breastfed and those who are picky eaters (defined widely and including food neophobia). Regarding breastfeeding duration and the association with food neophobia, the results are mixed. One longitudinal study found a negative association between breastfeeding duration and food fussiness (11), while Cassells et al. reported, from cross-sectional data in a randomized controlled trial, no correlation between breastfeeding duration and food neophobia, although this was not the aim of their study (12). One longitudinal study found that introducing complementary food at an earlier age was positively associated with fussy eating (11). No study has specifically addressed the associations between breastfeeding duration and food neophobia (10).

In Norway, there has been an extensive debate about whether children should be exclusively breastfed for 4 or 6 months (13, 14). The World Health Organization (WHO) recommendation is for 6 months (15). Being exclusively breastfed according to the WHO definition and recommendation entails no introduction to solids until 6 months of age. One of the main issues that has been discussed is whether prolonged exclusive breastfeeding would reduce later food variety and increase food neophobia (7). There is still little evidence on this relation. We therefore aimed to evaluate whether the duration of exclusive breastfeeding for 4 or 6 months and the duration of any breastfeeding are related to food neophobia in 1-year-old children.

## Methods

### Study design

The presented results are from the baseline study Barns Matmot 2.0, a web-based cluster randomized controlled trial in kindergartens to reduce food neophobia and promote healthy diets. The study protocol has been described elsewhere (16). The Norwegian Centre for Research Data approved the protocol (Ref. No. 49951).

### Participants

The recruitment of kindergartens started in May 2017 from all public and private kindergartens in four counties in Norway (Telemark, Oppland, Sør-Trøndelag, and Møre og Romsdal) that met the inclusion criteria (*n* = 1,043) of having children of the appropriate age (i.e. born in 2016). The following kindergartens were not included: those registered as “open” kindergartens where children and their parents attend together (*n* = 18), those registered with less than four children (*n* = 7), and those with children from 3 to 5 years old only (*n* = 12). The invitations were sent to the kindergarten managers by email and included detailed information about the study and a link to the study registration webpage. The kindergarten managers received one reminder email after a couple of weeks. Because few kindergartens (*n* = 32) registered for the intervention study initially, a random selection of kindergarten managers (*n* = 321) was additionally contacted by telephone and asked whether they had received and read the email. In total, 48 kindergartens registered for the study. Recruitment ended in October 2017.

**Fig. 1:**
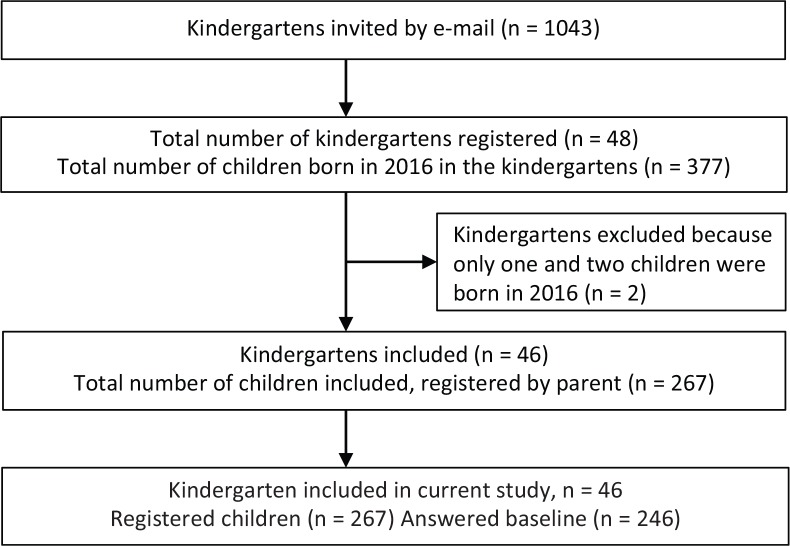
Flowchart of recruitment of kindergartens and children.

Before randomization, the pedagogical leaders in the participating kindergarten departments were asked to distribute an electronic invitation letter to the parents of children born in 2016, providing information about the study and a link to the registration webpage where parents could register their child for the study (www.uia.no/barnsmatmot2). Inclusion criteria for the children were that they had to be born in the year of 2016 and that at least one of the parents could read and understand Norwegian. Parents could register their child for the study from late August 2017 until the end of October, 2 weeks before the intervention started in the kindergartens in November 2017. In total, 267 children were registered by their parents for the study. An overview of the recruitment is given in the flow chart (Fig. 1).

The baseline questionnaires were sent to parents by email shortly after registration and had to be completed electronically before randomization and the start of the intervention. Of the 267 registered children, baseline data were only filled in for 246 children. Baseline data from these 246 form the basis for this study. During the recruitment period, two kindergartens were excluded (Fig. 1), and later, three intervention kindergartens withdrew from the intervention, leaving 43 kindergartens for the randomized controlled study. However, for the present cross-sectional analyses, we included data from the 46 kindergartens (Fig. 1) since parents had already agreed to participate and filled in questionnaires before their kindergarten withdrew from the intervention.

### Instruments

The parents filled in a questionnaire developed for this study that includes characteristics such as age, sex of the child and parent, and education of the father and mother. Furthermore, there were food frequency questions regarding the diets of both the child and parents.

Parents reported at what age the child had been introduced to different foods (porridge, canned dinners, fruits, fruit purees, bread, yoghurt, milk, formula, juice, and water) with response categories of “not had,” 0–2, 3, 4, 5, 6, 7, 8, 9, 10, 11, 12 or more months, and “do not know.”

Any breastfeeding was measured with the following question: “How old was your child when he or she stopped being breastfed?” The responses included never breastfed, 1 week, 2 weeks, 3–4 weeks, 2 months, 3 months, etc., up to 12 months, and then older than 12 months and still being breastfed. The responses were recoded in months, presented by 6 months or less, 7, 8, 9, 10, or 11 months, and 12 months or longer. Exclusive breastfeeding was calculated by first defining whether the child was breastfed in the given month, and then whether the child had been introduced to any food or drink other than breastmilk in the given month. To be categorized as exclusively breastfed, the child had to only receive breastmilk without being introduced to other food or drink.

Child food neophobia was measured with a six-item version of Pliner’s ten-item Child Food Neophobia Scale (CFNS) (17). The CFNS is a validated tool that uses parental reporting of child neophobia. The six-item version of CFNS is commonly used to measure food neophobia in young children and has been used with children as young as 2 years (17). In the present sample, the six items showed very good internal consistency, the Cronbach’s alpha of 0.88. The six items were as follows:1) “My child is constantly sampling new and different foods” (reversely scored), 2) “My child does not trust new foods,” 3) “If my child doesn’t know what a new food is, he or she won’t try it,” 4) “My child is afraid to eat new things he or she has never had before,” 5) “My child is very particular about the things he or she eats,” and 6) “My child will eat almost anything” (reversely scored). Responses ranged from “strongly disagree” to “strongly agree” on a 7-point scale. A CFNS score was computed with higher scores indicating higher levels of food neophobia. The total score ranged from 6 to 42. The CFNS items have been translated from English to Norwegian and then translated back to English (3). The CFNS was included in the parental questionnaire. Furthermore, parental food neophobia was assessed by the original 10-item Pliner score with different wording (e.g. I am constantly sampling new and different foods) with a score range from 10 to 70.

Highest completed education of both parents was asked for, with five response alternatives: less than 9 or 10 years of primary school, primary school, secondary school or high school, university/college 4 years or less, or university/college more than 4 years. This was recoded into high or low education, with having any university education defined as high education and no university education defined as low education.

### Statistics

Descriptive statistics with means, standard deviations (SD), and percentages were used to analyze the demographics of both the child and parent populations. Comparisons of food neophobia scoring between children being breastfed or exclusively breastfed and not breastfed at different ages were analyzed using two sample *t*-tests. Due to some skewness in the outcome variable food neophobia, we also performed non-parametric tests, independent samples Mann–Whitney *U*-test, showing the same results. We therefore only present the *t*-tests. Crude and adjusted linear regressions were performed regarding associations between breastfeeding duration (both any and exclusive breastfeeding) and food neophobia scoring. The breastfeeding variable was included as a dichotomous variable with a cut-off at the given month (6 to 12 months for any breastfeeding and 3 to 6 months for exclusive breastfeeding). Adjustments were done for maternal education and parental food neophobia scoring, according to the literature (3, 5). An additional analysis was conducted with any breastfeeding as a continuous variable (weeks of any breastfeeding). Such an analysis was not possible for exclusive breastfeeding as this variable lack details regarding 0–2 months of exclusive breastfeeding (introduction of solids was asked for from 0 to 2 months, then 3, 4, 5, etc. leaving the first category of exclusive breastfeeding less detailed than from 3 to 6 months). We also performed additional analysis of the association between food neophobia and breastfeeding with breastfeeding (any and exclusive) categories (presented in text). Because completion of the reported variables was mandatory in the current web-based questionnaire, there were no missing values. All analyses were performed using SPSS 25.0, and the significance level was set at *P* ≤ 0.05. The study size was calculated according to the primary outcome of the original randomized control trial (see protocol paper), and for this study, the baseline data are used.

## Results

The mean age of the children was 16.3 months (Table 1), ranging from 10 to 24 months. Forty-eight percent were girls, and all children participating were born in Norway. The mean parental age was 30.9 years, and 89% of those filling in the questionnaire were mothers. More than 90% of the parents were living together at the time of inclusion. The majority of the mothers had higher education, while more than 40% of fathers had higher education. Most parents were born in Norway.

**Table 1:** Baseline characteristics of participants included (mean [SD]) or *n* (%)

Characteristics	Values
**Child characteristics**	
Age (months), Mean (standard deviation [SD])	16.3 (3.1)
Gender female, *N* (%)	117 (48)
Ethnicity: Child born in Norway (%)	246 (100)
Breastfed ever	225 (92)
Breastfed at 4 months	201 (82)
Breastfed at 5 months	186 (76)
Breastfed at 6 months	174 (71)
Exclusively breastfed at 4 months	153 (62)
Exclusively breastfed at 5 months	60 (24)
Exclusively breastfed at 6 months	24 (10)
Child food neophobia at 16.3 months, mean (SD)	14.3 (7.1)
**Parent characteristics**	
Mean age in years (SD)	30.9 (5.4)
Gender female *N* (%)	218 (89)
Body mass index (kg/m^2^)	25.4 (4.4)
Parents living together (%)	232 (94)
Ethnicity: Mother born in Norway *N* (%)	225 (92)
Ethnicity: Father born in Norway *N* (%)	220 (89)
Mothers’ education high *N* (%)*	157 (64)
Fathers’ education high *N* (%)*	104 (42)
Parental food neophobia mean (SD)	23.6 (10.2)

All participants: *n* = 246.

* Higher education is defined as having any university/college education.

The mean child food neophobia score was 14.3 (SD 7.1), whereas the parental score was 23.6. Most of the children were still breastfed at 6 months (71%), while only 10% were exclusively breastfed until 6 months of age.

There was no significant difference in CFNS score at 16 months between those who were breastfed and those who were not at the respective time no longer breastfed for a shorter duration in the crude analysis. When adjusting for maternal education and parental food neophobia, which are both known to be related to child food neophobia (3, 5), those who were breastfed for 12 months or longer had significantly higher food neophobia score than those who were breastfed for a shorter period (Table 2). Food neophobia scoring was also significantly higher among those exclusively breastfed for 5 months or more compared to those exclusively breastfed for a shorter period in both crude and adjusted analyses (Table 3). In detail, this means that those who were breastfed for 12 months or longer and those who were exclusively breastfed for 5 months or longer were more prone to have a slightly higher level of food neophobia at 16 months of age compared to those who were breastfed for a shorter duration, with a score of about 1.5 to 2 points higher than those who were breastfed for a shorter duration.

**Table 2:** Differences in Child Food Neophobia Scale score at 16 months of age according to being breastfed (any breastfeeding) or not at different stages during the second half of infancy Crude and adjusted models

Age	Any breastfeeding	No breastfeeding	*P*^a^	Crude		Adjusted	
				*B*	*P*^b^	*B*	*P*^c^
**6 months**	*N* = 174	*N* = 72					
Child food neophobia	14.7 (7.4)	13.3 (6.4)	0.153	1.43 (−0.54, 3.40)	0.153	1.77 (−0.23, 3.77)	0.082
**7 months**	*N* = 162	*N* = 84					
Child food neophobia	14.8 (7.3)	13.3 (6.7)	0.099	1.54 (−0.35, 3.42)	0.109	1.84 (−0.08, 3.77)	0.060
**8 months**	*N* = 151	*N* = 95					
Child food neophobia	14.8 (7.3)	13.4 (6.8)	0.108	1.48 (−0.36, 3.32)	0.115	1.78 (−0.11, 3.67)	0.065
**9 months**	*N* = 129	*N* = 117					
Child food neophobia	14.8 (7.4)	13.6 (6.8)	0.198	1.17 (−0.62, 2.97)	0.200	1.47 (−0.37, 3.30)	0.117
**10 months**	*N* = 112	*N* = 134					
Child food neophobia	15.0 (7.5)	13.6 (6.8)	0.118	1.43 (−0.36, 3.23)	0.118	1.56 (−0.26, 3.39)	0.092
**11 months**	*N* = 95	*N* = 151					
Child food neophobia	15.2 (7.7)	13.7 (6.7)	0.107	1.51 (−0.33, 3.35)	0.107	1.74 (−0.10, 3.60)	0.064
**12 months**	*N* = 87	*N* = 159					
Child food neophobia	15.3 (7.9)	13.7 (6.7)	0.093	1.60 (−0.27, 3.47)	0.115	1.90 (0.12, 3.79)	0.049

a Two independent sample *t*-tests.

b Linear regression.

c Linear regression adjusted for maternal education and parental food neophobia.

**Table 3:** Differences in Child Food Neophobia Scale score at 16 months of age according to exclusive breastfeeding or not at 3, 4, 5, and 6 months or more of age (Crude and adjusted models)

Age	Exclusively breastfed	Not exclusively breastfed	*P*^a^	Crude		Adjusted	
				*B* (95% confidence interval [CI])	*P*^b^	*B* (95% CI)	*P*^c^
**3 months or more**	*N = 184*	*N = 62*					
Child food neophobia	14.2 (6.8)	14.4 (8.1)	0.233	−0.19 (−2.25, 1.89)	0.860	−0.09 (−2.16, 1.97)	0.926
**4 months or more**	*N* = 153	*N* = 93					
Child food neophobia	14.6 (6.9)	13.8 (7.5)	0.403	0.79 (−1.06, 2.64)	0.403	0.89 (−0.97, 2.76)	0.347
**5 months or more**	*N* = 60	*N* = 186					
Child food neophobia	15.9 (7.5)	13.7 (7.0)	0.046	2.11 (0.04, 4.20)	0.046	2.17 (0.11, 4.23)	0.039
**6 months or more**	*N* = 24	*N* = 222					
Child food neophobia	15.9 (7.8)	14.1 (7.1)	0.234	1.83 (−1.19, 4.85)	0.234	1.61 (−1.38, 4.60)	0.289

a Two independent sample *t*-tests.

b Linear regression.

c Linear regression adjusting for maternal education and parental food neophobia.

We also performed one analysis using any breastfeeding as a continuous variable, that is, weeks being breastfed in relation to CFNS scoring at 16 months. Crude results yielded B: 0.035 (95% confidence interval [CI]: −0.14, 0.084), p: 0.162, and adjusted results yielded B: 0.044 (95% CI: −0.006, 0.094), *P* = 0.087. Due to skewness because of three identified outliers, we performed sensitivity analysis without the three outliers (food neophobia scale: 38–42), with essentially the same results for exclusive breastfeeding, however not for any breastfeeding at 12 months where the *p*-value was above 0.05 (data not shown).

To further explore the relation between breastfeeding and food neophobia, we performed linear regression analysis using any breastfeeding (0, 1–3, 4–5, 6–8, 9–11, 12 months, and more than 12 months) and exclusive breastfeeding duration (exclusively breastfed 2 or less months, for 3, 4, 5, and 6 months or more) as categorical variables. There was no significant association between food neophobia and any breastfeeding Crude: *B* = 0.324 (95% CI: −0.127, 0.776), *P* = 0.159, and adjusted for parental food neophobia and maternal education Adjusted: *B* = 0.417 (95% CI: −0.049, 0.882), *P* = 0.079 in these analyses. There was no significant correlation between exclusive breastfeeding measured as a categorical variable of increasing duration: Crude: *B* = 0.203 (95% CI: −0.304, 0.710), *P* = 0.431, and adjusted for parental food neophobia and maternal education: *B* = 0.224 (95% CI: −0.285, 0.732), *P* = 0.387. In Table 4, we present the food neophobia score for those who stopped breastfeeding exclusively at 3, 4, 5, and 6 months and those who exclusively for less than 2 months. These numbers show that there is no linear relation between food neophobia and categories exclusive breastfeeding.

**Table 4:** Child food neophobia score measured at 16 months according to duration of exclusive breastfeeding

Exclusive breastfeeding	Food neophobia score (mean [SD])
Exclusively breastfed for less than 2 months (*n* = 62)	14.4 (8.1)
Exclusively breastfed for 3 months (*n* = 31)	12.5 (6.5)
Exclusively breastfed for 4 months (*n* = 93)	13.7 (6.3)
Exclusively breastfed for 5 months (*n* = 36)	15.8 (7.5)
Exclusively breastfed for 6 months (*n* = 24)	15.9 (7.7)

## Discussion

There was no significant difference in CFNS score at 16 months of age according to breastfeeding status reported at monthly intervals between 6 and 11 months of age, although CFNS scoring was numerically higher among children being breastfed at all timepoints. Those who were still breastfed at 12 months had significantly higher mean CFNS score than those with shorter duration of being breastfed. The same pattern was true for the relationship between being exclusively breastfed and later CFNS score. There was no significant difference in CFNS score at 16 months according to being exclusively breastfed or not at 4 months of age, but those who were still exclusively breastfed at 5 months or later had significantly higher CFNS score than those who were not. Our study adds to the literature regarding associations between breastfeeding mode and duration and the level of food neophobia with some indication that extended duration of both any breastfeeding for 12 months or more and exclusive breastfeeding for 5 months or more were weakly associated with higher scores of food neophobia at the mean age of 16 months. Previous literature is scarce regarding the specific outcome of food neophobia in relation to breastfeeding mode and duration (9, 12), with some literature showing negative associations between breastfeeding and food fussiness, and other literature showing the opposite or null findings (10, 18). Our study results are in favor of the latter.

Shim et al. found that children exclusively breastfed for 6 months were 75% less likely to be food neophobic in toddlerhood (at mean age of 3 years) compared to those who were not (9). Further, a Danish study found that children who had been exclusively breastfed until 5–6 months of age were less often categorized as picky eaters and had a higher vegetable intake than those only breastfed until 0–1 months of age (18). These results are not directly comparable to our findings due to the younger age of the children in this study and the majority of children being below the age at which food neophobia normally peaks.

A possible explanation for higher CFNS score with extended exclusive breastfeeding could be that the subgroup of children who are exclusively breastfed for 5 months or longer have been less exposed to a variety of foods at an early age, potentially leading to less willingness to try and accept new foods later. It is known from previous research that breastmilk varies in flavor depending on the maternal diet, thus exposing the infant to variation in taste even when fully breastfed (19). However, it could be that this variation is of less importance compared to exposure to food variety at this age and that a potential benefit of being breastfed on later food neophobia is negated by less sensory exposure to complementary foods.

Maier et al. (20) compared the acceptance of new foods between formula-fed and breastfed infants when given a variety of foods at different frequencies. They found that both being breastfed as opposed to being formula fed and given a variety of foods early during weaning rather than being given a certain food often resulted in better acceptance of new foods when measured some weeks after the intervention (20). This study illustrates the complicated relation between milk feeding mode, complementary feeding and food acceptance. Several predictors for food neophobia exist, and the lack of early exposure to a variety of foods is one of these predictors (5, 10).

The transmission of taste compounds from the mother’s diet through breastmilk to the infant has been observed; however, the magnitude of such transmission could vary widely from mother to mother and can differ according to what food is eaten (21). The taste exposure through mother’s milk is therefore likely to be variable (22). Research suggests that exposure to the actual foods has a more robust effect on acceptance than the taste transmitted through breastmilk (22). Harris and Coulthard (22) suggested that a combination of breastfeeding with the timely introduction of complementary foods has the best effect on the acceptance of new foods and should be the best strategy for developing infant acceptance of foods, such as fruit and vegetables.

A strength of our study is that it is performed in a country with high breastfeeding rates compared to other countries in which these associations have been previously examined. More than 70% of the children were breastfed at 6 months, and 10% were exclusively breastfed at 6 months.

It is worth noting that the overall level of food neophobia is low in this study, which is however in line with Cassell et al.’s (12) study of 2-year-olds in Australia. The distribution of the food neophobia score was somewhat skewed toward lower score, indicating that most children were not neophobic as yet at the time when neophobia was assessed. The peak of food neophobia is around 2 years of age; therefore, the observed level is expected. However, one should note that the food neophobia scale was originally developed for 5–8-year-old children, and even though it has been used in 2-year-olds, it has not previously been used in younger children. The scale was developed 25 years ago, and since the connotations of ‘novel’ food has changed for most people in the Western world (23), new methods should probably be applied in future studies. Despite limitations with the scale, the internal consistency was good, and we found a small difference in food neophobia at 16 months according to breastfeeding duration. The observed numerical differences in CFNS according to breastfeeding status at different time points varied between 1.5 and 2 points. Whether a difference of this magnitude has any predictive value regarding later food neophobia, or fussiness, is difficult to say. Helland et al. (3) have shown a linear relationship between neophobia and healthy food items, which could mean that any positive change in neophobia scoring could improve diet. In addition, in a public health perspective even small differences may be of relevance when the exposure is common. In addition, one could speculate that the association would be stronger if food neophobia had been assessed later, for example, around or after the age of two when food neophobia normally peaks. Future studies should therefore include longer follow-up.

Given the cross-sectional nature of the data, we cannot exclude the possibility of reverse causation, that is, that higher child food neophobia or fussiness may have led to longer duration of breastfeeding in our sample. If this were the case, breastfeeding mode and duration might not have any causal relationship with food neophobia, either positive or negative.

Residual or unmeasured confounding of the observed association by factors associated with both breastfeeding duration and food neophobia, such as parenting style, feeding practices, and hereditary factors, cannot be excluded. A further limitation of our study is the potentially low generalizability because of low participation rate. Due to the nature of the study, we lack information about those who did not register for participation. We also lack information about those who registered and did not fill in the baseline questionnaire (*n* = 21). Compared to national numbers, our participants had slightly higher education than Norwegian parents in general (24). Given that breastfeeding and breastfeeding duration are related to maternal education, this may have affected our results (25). Those participating might breastfeed longer and be more aware of dietary guidelines than the general public. If so, one could expect that a clearer relation would be found with a greater diversity in breastfeeding duration and child diet. On the other hand, the age of parents was comparable with the mean age of mothers of 1-year-olds in Norway. Further, the geographic diversity and the diversity in the size and type of kindergartens from which the children were recruited may enhance the generalizability of our findings. The data were self-reported, and breastfeeding was assessed in retrospect when the child had reached 16 months of age. For the latter, the maternal memory of the duration of breastfeeding seems to be quite high (26).

## Conclusion

Still being breastfed at 12 months of age was associated with a slightly higher CFNS score at 16 months compared to shorter breastfeeding duration. Similarly, being exclusively breastfed at 5 months or longer was associated with slightly higher CFNS score at 16 months of age compared to shorter exclusive breastfeeding. No other associations between breastfeeding duration and food neophobia were found. As data were derived from a cross-sectional study, our findings should be interpreted with caution.

## Data Availability

We are working on several papers on the same data material and do not wish to share our material before we have thoroughly analyzed data.
